# Prevalence of diarrheal and its associated factors among children aged under-five years in Amhara Regional State, Ethiopia: a cross-sectional study

**DOI:** 10.1038/s41598-024-76540-5

**Published:** 2024-11-18

**Authors:** Gebru Gebremeskel Gebrerufael, Brhane Gebrehiwot Welegebrial, Kidanemariam Alem Berhie, Bsrat Tesfay Hagos, Mehari Gebre Teklezgi

**Affiliations:** 1 Department of Statistics, College of Natural Science, Adigrat University, Adigrat, Ethiopia; 2 Department of Pharmacy, College of Medicine and Health Sciences, Adigrat University, Adigrat, Ethiopia; 3 Department of Biostatistics, School of Public Health, College of Health Science, Mekelle University, Mekelle, Ethiopia; 4 Department of Statistics, College of Natural Science, Mekelle University, Mekelle, Ethiopia

**Keywords:** Amhara Regional State, Diarrhea, Ethiopia, Logistic regression, Risk factors, Diseases, Medical research, Risk factors

## Abstract

Diarrhea is a major health problem in Ethiopia, and the Amhara Regional State is experiencing the highest prevalence rate of diarrhea. Therefore, this study was designed to assess the prevalence and risk factors attributed to diarrhea among children under-five in the Amhara Regional State. The study employed descriptive statistics and multivariable logistic regression analysis to identify the main statistically significant associated factors of diarrhea using Stata version 14. The overall prevalence rate of experiencing diarrhea was 17.7% [95% CI: 15.4, 20.2] in the Amhara Regional State. The multivariable logistic regression model analysis indicated that the risk factors are fourth and above birth order [AOR = 2.95, 95% CI (1.483, 5.85)], gender of child (being male) [AOR = 1.66, 95% CI (1.144, 2.41)], no education level of mother’s [AOR = 4.7, 95% CI (1.01, 22.0)], being age of child 7–11 [AOR = 3.33, 95% CI (1.58, 7.01)] and 12–23 month [AOR = 2.17, 95% CI (1.11, 4.22)], mother’s age at 15–19 years [AOR = 12.2, 95% CI (2.986, 50.2)], mother’s age at 20–24 years [AOR = 6, 95% CI (1.758, 20.8)], and mother’s age at 25–29 years [AOR = 4.48, 95% CI (1.41, 14.2)], and five and greater than family size [AOR = 0.53; 95% CI (0.343, 0.84)] were statistically significant associated with a higher risk of diarrhea. The prevalence of diarrhea in the Amhara Regional State was higher than that at the national level. The study identified gender of a child, education level of the mother’s, birth order, family size, mother’s age, and child’s age as risk factors for diarrhea in children under-five.

**Supplementary Information:**

The online version contains supplementary material available at 10.1038/s41598-024-76540-5.

## Introduction

Diarrhea is one of the most challenging public health problems and is responsible for killing approximately 8% (525,000) of children every year in the world^1^. Globally, 1.7 billion children have diarrhea incidents occurring among children under 5 years of age annually^2^. Among children under five years old, diarrhea accounts for 17% of all deaths^3^. Similarly, diarrhea is a major health problem in Ethiopia^4^. For this reason, in Ethiopia, diarrhea is the leading cause of death for children under the age of 5 years^5^.

The risk factors associated with diarrhea are child age, education level of the mother’s, residence, and gender of the child^1,6^. In Ethiopia, infants and children under 5 years of age show a continuous declining trend in the previous 15 years. However, the prevalence rate of diarrhea in Ethiopia was reduced from 18% in 2005 to 13% in 2011 and to 12% in 2016, according to the Ethiopia Demographic and Health Surveys (EDHS). Based on the 2016 EDHS, the Amhara Regional State has experienced a higher prevalence rate of diarrhea of 13.7% compared to the national average rate of 11.8%^7^.

Despite a decline in deaths among children under five, diarrhea prevalence and mortality remain high in Ethiopia. Most studies focus on national data, which may overlook regional specifics crucial for policymakers. To address this gap, we conducted a cross-sectional analysis of the 2016 EDHS to identify major diarrhea risk factors and prevalence in the Amhara Regional State, considering various socio-demographic, economic, and environmental factors^7^.

## Methods

### Study design, data source, period, and study setting

This study was conducted using a retrospective cross-sectional study design from the 2016 EDHS report. The data collection process was carried out via the Central Statistics Agency (CSA), the Ethiopian public health institute, and the Ministry of Health from January to June 2016. The source of funding was obtained from the United States (UN) agency for international development. The major purpose of this investigation is to supply detailed information on fertility, family planning, child, adult, and mother mortality, maternal and child health, diarrhea infection, and knowledge of HIV/AIDS^7^.

The 2016 Ethiopia Demographic Health Survey (EDHS) report has three questionnaires. These were women’s, households, and men’s questionnaires.

All women aged 15–49 years and who had either been stable residents of the chosen households or had lived in the household at least one night earlier in the study were qualified for the face-to-face interview. The dataset was gathered by directly interviewing women who met the eligibility criteria. Overall, of 15,683 females of reproductive age (15–49 years) and 12,688 men aged 15–59 years in 16,650 households interviewed in the 2016 EDHS, 1,902 households were from the Amhara Region State^7^.

It is also bordered by Tigray Regional State in the north, Afar in the east, Oromiya in the south, Benishangul-Gumiz in the southwest, and Sudan in the west. The 2007 Ethiopian population and housing censuses reported that the Amhara Regional State had a total population number of 17,214,056 (23.3%), of which 8,636,875 (50.2%) were males. Approximately 87.4% (15,101,836) of the total population are rural residents^8^.

### Study population and sampling methods

**Fig. 1:**
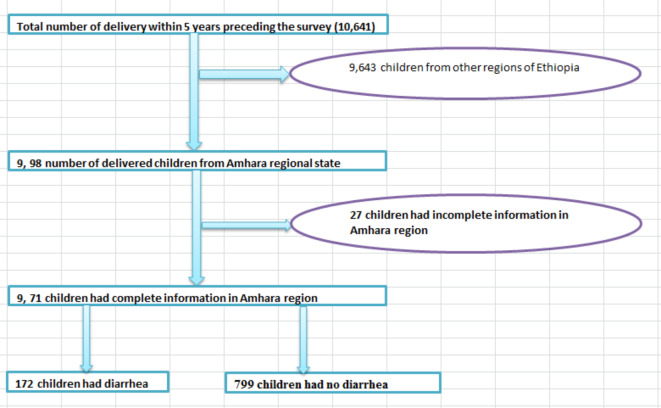
Schematic presentation of children under-five with diarrhea included in the study.

The survey had information on a range of socioeconomic and demographic predictor factors of the population nationwide. Amhara regional state was selected because the 2016 EDHS report revealed that it had one of the highest rates of diarrhea in the country. All women aged 15–49 years who had at least one child in the five years before the two weeks preceding the survey were eligible for participation in each cluster. In the region, 998 births were reported in the previous five years preceding the 2016 EDHS survey conducted. Next, children with detailed information on diarrhea during the last five years were presented in the full 2016 EDHS report^7^. Finally, the study included 971 children under five, comprising 172 with diarrhea and 799 without, all having complete data on the considered predictor factors for the final analysis [see Fig. 1].

### Operational definitions

Diarrhea was defined as having as much as 3 and greater loose (liquid stools) over a 24-hour period, as informed by the mother of the child’s at any point during the two weeks preceding the interview^1,9,10^.

**Unprotected water source**: while individuals (people) use water for drinking from a river, pond, well, or unprotected spring^10,11^.

### Variables of the study

#### Outcome variable

The outcome variable for this study was the diarrhea of children under five years, which was a dichotomous (binary) outcome variable coded as “0” for no and “1” for yes. Such that

$${\text{Y}}_{i} = \left\{ {\begin{array}{*{20}l} {1,} & {\text{if}\:i^{{th}} \:\text{child's had experienced diarrhea}\:\left( {{\text{Yes}}} \right)} \\ {0,} & {\text{if}\:i^{{th}} \:\text{child's had not experienced diarrhea}\:\left( {{\text{No}}} \right)} \\ \end{array} } \right.$$

### Independent variables

The risk factors for diarrhea in this study were selected from prior studies. Mother’s and child demographic characteristics explored from the theoretical perspective, datasets, and different literature on diarrhea are presented in Table 1.

**Table 1:** Description of explanatory (independent) variables in the study.

Variables	Categories/coded value
Family size	0 = Less than five, 1 = Five and greater than
Mother’s age (in year)	0 = 45–49, 1 = 40–44, 2 = 35–39, 3 = 30–34, 4 = 25–29, 5 = 20–24, 6 = 15–19
Place of residence	0 = Urban, 1 = Rural
Education level of mother’s	0 = Higher, 1 = Secondary, 2 = Primary, 3 = No education
Water drink source	0 = Protected water, 1 = Unprotected water
Toilet facility	0 = Pit or flash toilet, 1 = No facility
Gender of child	0 = Female, 1 = Male
No. of children < 5 years in household	0 = 2 or less, 1 = 3 and above
Duration of breast feed < 6 month	0 = No, 1 = Yes
Birth order of child’s	0 = First, 1 = Second, 2 = Third, 3 = Fourth and above
Age of child (in month)	0 = 0–6, 1 = 7–11, 2 = 12–23, 3 = 24–35, 4 = 36–47 and 5 = 48–59

### Data processing and analysis

The collected dataset was extracted, edited, checked for completeness and consistency, and coded in SPSS version 20. Once entered and checked, the dataset was exported to Stata version 14 for cleaning and further analysis (see Additional File 1). Both descriptive and inferential statistics were used in the final analysis. A bivariable logistic regression model was performed to assess the effect of individual association risk factors on diarrhea, and those variables were included in the multivariable logistic regression model analysis to identify the major predictors of diarrhea.

Since the VIF value for all predictor variables is < 10, there is no multicollinearity problem (see Additional File 2). Moreover, goodness of fit to the final model was cross-checked via Hosmer and Lemeshow, and a good fit was found. The strength of association between the risk factor variables and the response variable was measured using the adjusted odds ratio (AOR) and significant variables at the 5% level of significance (p-value < 0.05).

### Ethical consideration

In order to use the 2016 EDHS dataset from the DHS program for the current study, a formal consent letter was discovered. The dataset was also given IRB approval for public use without individual or household identity. As a result, the participants’ identities were kept secret. Furthermore, this dataset was exclusively used for the current study in accordance with the DHS program discipline, rules, and regulations. However, access to the study’s data set requires legal registration at https://dhsprogram.com/data/available-datasets.cfm and the creation of a persuasive letter outlining the project’s descriptive goal for the DHS program.

## Results

### Descriptive statistics analysis of children under 5 years of age

A total of 971 children were included in the final data analysis. The prevalence rate of diarrhea among children under 5 years of age was found to be 17.7% [95% CI: 15.4, 20.2] in the two-week surveillance period. The majority of 691 (71.2%) mothers had no educational status. Of the total participants, 527 (54.3%) had no toilet facility, and 789 (81.3%) didn’t have a water source. The majority of 509 households (52.4%) had less than five people. Among the total participants, 881 (90.7%) lived in rural areas. More than half (52.7%) of the children were males. From the study participants, the majority (495 (51%)) of the children were fourth and above the birth order of their family (see Table 2).

**Table 2:** Summary of sociodemographic and clinical predictors of children under 5 years of age in the Amhara Regional State, Ethiopia (*N* = 971).

Variables	Categories	Diarrhea status of children		
		Yes, *N* (%)	No, *N* (%)	Total
Family size	Less than five	112(11.5%)	397(40.9%)	509(52.4%)
	Five and greater than	60(6.2%)	402(41.4%)	462(47.6%)
Mother’s age (in year)	45–49	4(0.41%)	41(4.2%)	45(4.6%)
	40–44	9(0.93%)	81(8.3%)	90(9.3%)
	35–39	26(2.7%)	156(16.1%)	182(18.7%)
	30–34	29(3.0%)	164(16.9%)	193(19.9%)
	25–29	54(5.6%)	221(22.8%)	275(28.3%)
	20–24	34(3.5%)	118(12.2%)	152(15.7%)
	15–19	16(1.7%)	18(1.8%)	34(3.5%)
Place of residence	Urban	16(1.7%)	74(7.6%)	90(9.2%)
	Rural	156(16.1%)	725(74.7%)	881(90.7%)
Education level of mother’s	Higher	2(0.2%)	32(3.3%)	34(3.5%)
	Secondary	8(0.8%)	62(6.4%)	70(7.2%)
	Primary	25(2.6%)	151(15.6%)	176(18.1%)
	No education	137(14.1%)	554(57.1%)	691(71.2%)
Water drink source	Protected water	33(3.4%)	149(15.4%)	182(18.7%)
	Unprotected water	139(14.3%)	650(66.9%)	789(81.3%)
Toilet facility	Pit or flash toilet	68(7.0%)	376(38.7%)	444(45.7%)
	No facility	104(10.7%)	423(43.6%)	527(54.3%)
Gender of child	Female	61(6.3%)	398(41%)	459(47.3%)
	Male	111(11.4%)	401(41.3%)	512(52.7%)
No. of children < 5 years in household	2 or less	165(17.0%)	731(75.3%)	896(92.3%)
	3 and above	7(0.7%)	68(7.0%)	75(7.7%)
Duration of breast feed < 6 month	Yes	48(4.9%)	217(22.4%)	265(27.3%)
	No	124(12.8%)	582(59.9%)	706(72.7%)
Birth order	First	31(3.2%)	132(13.6%)	163(16.8%)
	Second	25(2.6%)	149(15.3%)	174(17.9%)
	Third	22(2.3%)	117(12.1%)	139(14.3%)
	Fourth and above	94(9.6%)	401(41.3%)	495(51%)
Age of child (in month)	0–6	16(1.7%)	98(10.1%)	114(11.7%)
	7–11	28(3.0%)	56(5.8%)	84(8.7%)
	12–23	49(5.0%)	140(14.4%)	189(19.5%)
	24–35	32(3.3%)	144(14.8%)	176(18.1%)
	36–47	27(2.8%)	169(17.4%)	196(20.2%)
	48–59	20(2.1%)	192(19.8%)	212(21.8%)

**Fig. 2:**
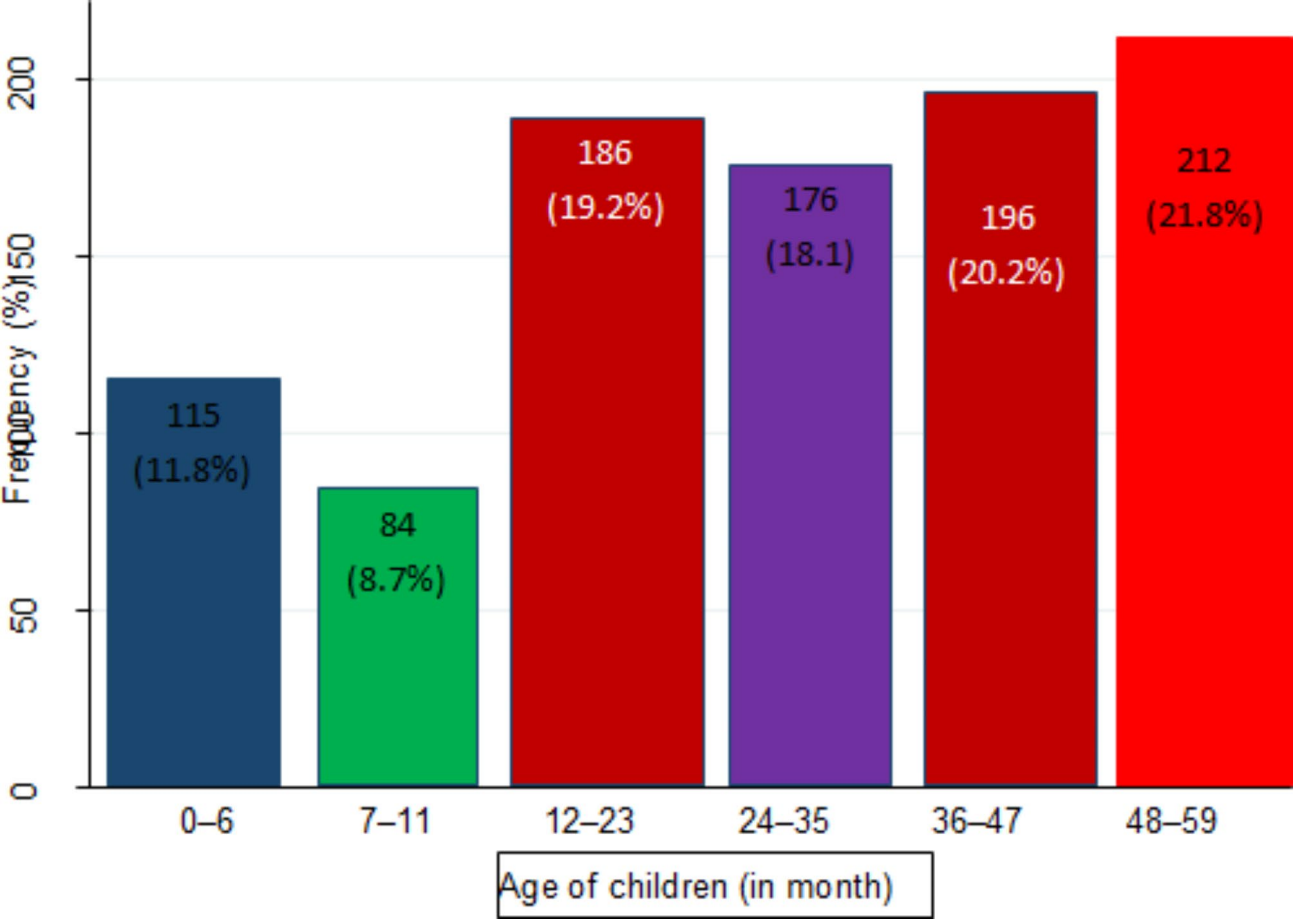
Diarrhea infection with respect age among under-5 year’s children in Amhara Regional State, Ethiopia, 2016.

The highest prevalence rate of diarrhea 212 (21.8%) occurred between children aged 48–59 months (see Fig. 2).

### Assessment of parsimonious model fitness of the model

We checked the overall goodness of fit using the Omnibus Tests of Model (OTM) and Hosmer-Lemeshow tests. Consequently, the Omnibus Tests of Model provided a X^2^ (24) = 117.63 and p-value < 0.001, which would imply goodness-of-fit found for the model. Similarly, the Hosmer and Lemeshow Test found the observed data was better explained by the model [X^2^ (8) = 8.25 and p-values = 0.409].

### Factors associated with the risk of diarrhea among children under-5 years of age

In the multivariable logistic regression model analysis, mother’s age, education level of mother’s, family size, gender of child, and age of child were significantly associated with the risk of diarrhea (P-value < 0.05).

The result from the multivariable logistic regression model analysis shows that the odds ratio of developing diarrhea between the children aged 7–11 months and 12–23 months were 3.33 and 2.17 times more likely to develop diarrhea [AOR = 3.33, 95% CI (1.58, 7.01)], [AOR = 2.17, 95% CI (1.11, 4.22)] than 0–6 months, respectively.

Moreover, being the fourth and above birth order $\:[\text{A}\text{O}\text{R}=$2.95, 95% CI (1.483,5.85)], being male child $\:[\text{A}\text{O}\text{R}$=1.66,95% CI (1.144, 2.41)], having no education level of mother’s [AOR=4.7, 95% CI (1.01, 22.0)], being mothers at age of 15–19, 20–24, and 25–29 years with their [AOR=12.2, 95% CI (2.986, 50.2)], [AOR=6, 95% CI (1.758, 20.8)], and [AOR=4.48,95% CI (1.41,14.2)], respectively, were significant predictors of diarrhea (see Table 3).

**Table 3:** Bivariable and multivariable analysis of risk factors for diarrhea among children under 5 years of age in the Amhara Regional State, Ethiopia (*N* = 971).

Variables	Bivariable logistic regression				Multivariable logistic regression			
	95%CI for COR				95%CI for AOR			
	COR	Lower	Upper	*P*-value	AOR	Lower	Upper	*P*-value
Family size (ref.= less than five)								
Five and greater than	0.28	0.2	0.4	< 0.001*	0.53	0.343	0.84	0.007*
Mother’s age (in year)(ref.= 45–49)								
40–44	1.14	0.33	3.91	0.837	1.1	0.309	3.94	0.879
35–39	1.71	0.56	5.22	0.343	1.84	0.59	5.73	0.292
30–34	1.81	0.6	5.43	0.289	2.16	0.692	6.72	0.186
25–29	2.5	0.85	7.36	0.092	4.48	1.41	14.2	0.010*
20–24	2.95	0.99	8.85	0.052	6	1.758	20.8	0.005*
15–19	9.12	2.65	31.3	< 0.001*	12.2	2.986	50.2	0.001*
Place of residence (ref.= urban)								
Rural	1	0.57	1.77	0.987	0.76	0.362	1.61	0.467
Education level of mother’s (ref.= higher)								
Secondary	2.06	0.41	10.3	0.377	2.15	0.406	11.4	0.368
Primary	2.65	0.6	11.7	0.2	2.16	0.451	10.4	0.336
No education	3.96	0.93	16.9	0.061	4.7	1.01	22	0.049*
Water drink source (ref.= protected water)								
Unprotected water	0.96	0.64	1.45	0.87	0.84	0.492	1.42	0.511
Toilet facility (ref.= Pit (flash toilet)								
No facility	1.36	0.97	1.9	0.073	1.13	0.762	1.67	0.544
Gender of child (ref.= female)								
Male	1.81	1.29	2.52	0.001*	1.66	1.144	2.41	0.007*
No. of children < 5 years in household (ref.=2 or less)								
3 and above	0.45	0.2	1.01	0.053	0.49	0.204	1.19	0.111
Duration of breast feed < 6 month (ref.= no)								
Yes	0.96	0.66	1.39	0.842	0.78	0.496	1.22	0.285
Birth order (ref.= first)								
Second	0.71	0.4	1.26	0.253	0.91	0.479	1.75	0.784
Third	0.8	0.44	1.47	0.468	1.1	0.528	2.25	0.812
Fourth and above	1	0.64	1.57	0.994	2.95	1.483	5.85	0.002*
Age of child (in month)(ref.= 0–6)								
7–11	3.06	1.51	6.2	0.002*	3.33	1.58	7.01	0.002*
12–23	2.14	1.15	3.98	0.016*	2.17	1.11	4.22	0.022*
24–35	1.36	0.71	2.61	0.355	1.25	0.618	2.54	0.531
36–47	0.98	0.5	1.91	0.949	1.1	0.511	2.2	0.877
48–59	0.64	0.32	1.28	0.209	0.63	0.288	1.38	0.247

COR: Crude Odds Ratio, AOR: Adjusted Odds Ratio, * Significant at 5% level of significance.

## Discussion

This study was conducted to identify the important risk predictors and prevalence rate of diarrhea among children under 5 years of age in the Amhara Regional State, Ethiopia. The EDHS of 2016 revealed that diarrhea in the Amhara Regional State had a higher prevalence rate than that at the country level. The results of this investigation indicated that the prevalence rate of diarrhea among children under 5 years of age was 17.7% [95% CI: 15.4, 20.2]. This finding was consistent with a study found in the Jabithennan district, Northwest Ethiopia, Farta Wereda, North-West Ethiopia, Bahir Dar Zuria district, North-West Ethiopia, and North Gondar zone, Ethiopia, 21.5%, 16.7%, 20%, and 21.1%, respectively^6,10–12^. In contrast, it was less than the study conducted in the Hadaleala district, Afar Region, North-East Ethiopia, and in the Harena Buluk Woreda, Oromia Region, South East Ethiopia, 31.3% and 28.4%, respectively^3,13^. This variance might be due to seasonal trends in diarrhea, the period of investigation, and the method of data collection.

Children under 5 years of age in the 7–11 and 12–23 month age groups were at higher risk of emerging diarrhea than children aged less than 6 months. This result was similar to the results found in the study shown in the Hadaleala district, Afar Region, North-East Ethiopia, Farta Wereda, North-West Ethiopia, and Kenya^1,10,13^. This may be due to the child’s beginning to walk at 6 months, decreased mother’s antibodies, and the risk of feeding contaminated resources. At this age, the child begins additional supplementary feeding that may increase the risk of contamination with nutrients and water. The less manifestation of diarrhea during the age 0–6 months definitely shows the protective effect of exclusive breast feeding of children and less exposure to dirtied agents in the first seven months of life.

This study showed that diarrhea in the birth order fourth and above was more affected compared with the first birth order. This finding was consistent with a study in the Jigjiga district, Somali Region, Eastern Ethiopia^14^. This result might be because of the low attention of mothers, who become less conscious of their children^1,11^.

Similarly, this study illustrated that children whose mothers had no educational level were 4.7 times more likely to have diarrhea [AOR = 4.7, 95% CI (0.995, 22)] than mothers with a higher educational level. This finding is in line with the Hadaleala district, Afar Region, North-East Ethiopia, and Pawi Hospital, Northwest Ethiopia^13,15^. This may be because mothers have low awareness of the influences of sanitary experience, the child’s feeding system, and hygiene practices, which are considered significant predictors of children’s diarrhea.

Another essential demographic risk factor significantly associated with diarrhea was the gender of the child. Being male, children were at a higher risk of diarrhea. In contrast, a study in the literature claimed that this could be due to genetic variances among male and female children^16,17^. Furthermore, an additional study carried out in Burundi indicated that gender didn’t play a significant role in relation to diarrhea prevalence rates for different age groups^18^.

Moreover, mothers’ ages of 25–29, 20–24, and 15–19 years were also significantly associated with diarrhea in their children. The adjusted odds ratio (AOR) of the age group of mothers 25–29, 20–24, and 15–19 [AOR = 4.48, 95% CI (1.41, 14.2)], [AOR = 6, 95% CI (1.758, 20.8)], and [AOR = 12.2, 95% CI (2.986, 50.2)] were higher than those of the age group 45–49, respectively. This suggests that diarrhea decreases significantly with increasing age. Besides, that shows that women of older ages were good at protecting against diarrhea due to their mature enough capacity to experience how to care for their children.

### Strengths and limitation and of the study

The utilization of nationally representative survey datasets by the authors of this study enhances the findings drawn at the national level. Due in significant part to the retrospective research design of the interview and the high percentage of missing values in the 2016 EDHS data, recall bias may be present in the 2016 EDHS, which relies heavily on respondents’ self-reports. Furthermore, the study excluded a number of important predictor variables, including the age of the pregnant mother. The investigators should be informed that multilevel model analysis is preferred to better account for these limitations when their dataset is structured in a hierarchical manner, as in the case of the 2016 EDHS data set.

## Conclusions

In general, the prevalence of diarrhea in children under 5 years of age is quite high (17.7% [95% CI: 15.4, 20.2]) in the Amhara regional state; especially in children aged 7–11 and 12–23 months of age and whose mothers are aged 15–19, 20–24, and 25–29 years of age. Based on multivariable logistic regression model analysis, the risk factors for diarrhea in children were mother’s age, education level of mother’s, family size, gender of child, and age of child were significantly predictors of diarrhea (P-value < 0.05). Therefore, to lower the incidence of diarrheal illness in children under five, public health initiatives and concerned body service providers should give more attention to maternal education opportunities.

## Electronic supplementary material

Below is the link to the electronic supplementary material.

Supplementary Material 1

## Data Availability

The dataset will be obtainable based on request from the corresponding author on reasonable request.

## Abbreviations

CSA: Central Statistical Agency
CI: Confidence interval
EDHS: Ethiopian Demographic and Health Survey
AOR: Adjusted odds ratio
COR: Crude odds ratio
Ref: Reference
UN: United States
WHO: World Health Organization
