# Supplementary material for: Prevalence of diarrheal and its associated factors among children aged under-five years in Amhara Regional State, Ethiopia: a cross-sectional study
Source: Sci Rep. 2024 Nov 18;14:28481. doi: 10.1038/s41598-024-76540-5 (PMC11574280; doi:10.1038/s41598-024-76540-5)
Supplement: Supplementary file 1 — Supplementary Material 1 [file 41598_2024_76540_MOESM1_ESM.docx]

**Additional file 1.** Syntax in Stata for multivariable logistic regression model analysis (Stata version 14).

**Appendix**

**Stata codes for data analysis**

**Multivariable logistic regression analysis**

**/*All risk factors bi-variable logistic regression were entered into multi-variable logistic to control confounding effects*/**

logistic Diarrhea_Status i.Women_ageinyear i.place_of_residence i.mothers_education i.water_Drink_source i.toilet_facility i.family_size i.No_of_children_5inhousehold i.breast_feed_less6month i.Birth_order i.Gender_of_child i.age_of_child_inmonth

/***Multi-collinearity test was performed to assess the existence of correlation among the predictor variables***/

vif, uncentered

/***Goodness-off- fit to the final model was checked by Hosmer and Lemeshow and LRT and was found fit** */

estat gof

estat ic

**Additional file 2:** **Table 1** shows the multicollinearity of predictor variables.

|  |  |
| --- | --- |
| **Variables** | VIF |
| **Family size** (ref.= less than five) |  |
| Five and greater than | 3.02 |
| **Mother’s age** (in year)(ref.= 45–49) |  |
| 40–44 | 1.67 |
| 35–39 | 4.6 |
| 30–34 | 5.45 |
| 25–29 | 3.87 |
| 20–24 | 3.79 |
| 15–19 | 2.39 |
| Place of residence (ref.=Rural) |  |
| Urban | 5.69 |
| **Education level of mother’s** (ref.= higher) |  |
| Secondary | 9.64 |
| Primary | 5.23 |
| No education | 2.52 |
| Water drink source (ref.= unprotected water) |  |
| Protected water | 7.45 |
| Toilet facility (ref.= No facilit) |  |
| Pit (flash toilet) | 2.57 |
| **Gender of child** (ref.= male) |  |
| Female | 2.18 |
| No. of children <5 years in household (ref.=3 and above) |  |
| 2 or less | 1.21 |
| Duration of breast feed <6 month (ref.= yes) |  |
| No | 4.58 |
| **Birth order** (ref.= first) |  |
| Second | 2.28 |
| Third | 2.41 |
| Fourth and above | 8.13 |
| **Age of child** (in month)(ref.= 0–6) |  |
| 7–11 | 1.67 |
| 12–23 | 2.49 |
| 24–35 | 2.4 |
| 36–47 | 2.64 |
| 48–59 | 2.82 |
| **Mean VIF** | **4.59** |
